# Notes and News

**Published:** 1897-10-02

**Authors:** 


### Notes and News.

(Collected and Communicated.)

Mr. George Hanbury, the treasurer of the Paddington Green Children's Hospital, has generously given a donation of £100 towards paying off a debt of £1,900 due to the hospital bankers.

The dinner to be given to Sir Samuel Wilks, Bart., at the Hotel Metropole on October 6th, to congratulate him on receiving his new honour, is sure to be most popular and well attended. The secretary, Dr. Newton Pitt, 15, Portland Place, W., is receiving the names of intending participators.

The plague again appears to be gaining ground in India, Surat; Thana, Poona, Satara, Nasik, Ratnagiri, and other places all being more or less affected. The local press is expressing uneasiness lest the frontier operations should attract the medical service, and thus leave the sick unattended.

Dr. Marsden, medical officer for Birkenhead, brought before the Leeds Sanitary Congress the subject of theatrical advertisements depicting acts of violence and bloodshed. He thought them likely to excite ill-balanced minds, and produced some of the class of placards to which his objections referred, saying that he felt certain that many crimes could be traced to these degrading advertisements.

We are glad to observe that the governors of the Northampton General Infirmary, at the instigation of the honorary medical staff and committee, are promoting a testimonial to Mr. G. P. Bennett, who for 39 years has rendered invaluable services to the infirmary in the office of secretary, at a very inadequate salary. It is felt that the governors and subscribers should mark their appreciation of Mr. Bennett's services on his retirement, which was recently announced. We cordially support this view, and hope that the subscriptions may amount to a sufficient sum to enable the testimonial to take a substantial form, of real value and permanent interest.

Guy's Hospital has received the sum of £4,000 from a lady, who desires to remain anonymous, for the purpose of endowing four beds in perpetuity. This gift will form part of the re-endowment fund.

The trustees of the Sir Morell Mackenzie Memorial Fund have handed over to the Throat Hospital, Golden Square, £1,023 towards the building of the memorial wing of the extension now being carried out.

The Royal National Lifeboat Institution have received a donation of £1,000 from Mr. James Coats, jun., Paisley, who states that this gift is made as a protest against the attacks to which the affairs of the society have lately been subjected.

After the State service at Stockholm, held in commemoration of King Oscar of Sweden's reign of 25 years, various deputations presented offerings from the nation amounting to 220,000 kroners. The King has decided to devote the offering to some method of combating tubercular disease.

Dr. Angus has been elected Superintendent of the Aberdeen Royal Infirmary. The post was held in an honorary capacity by Miss Lumsden, who has resigned. Dr. Angus had a distinguished career as a student, and held several good appointments, the last being senior medical assistant at the Aberdeen Lunatic Asylum.

On October 19th, at half-past seven p.m., the Archbishop of York will preach at St. Paul's Cathedral to the members of the medical profession. The service, which has been initiated by the Guild of St. Luke, is likely to be a brilliant one, as all doctors who are graduates are asked to attend in academical robes. At the corresponding service last year upwards of 1,100 medical men were present, and the space under the dome of the cathedral was half filled with those in academical dress. The music will be rendered by a choir of over 200 voices, provided by the London Gregorian Association, so that the whole service will constitute a unique ecclesiastical event. Admission to the space under the dome will be by tickets only.

Surgeon-Major Barry, who has been investigating in Poona, gives a most shocking description of the sanitation of the city. His report says that, under the present municipality, the city has fallen into "the groove of chaos, of incompetency, inefficiency, waste, absence of check, and wanton disregard of public obligations towards the poorer citizens." "The Brahmins of Poona have had for years an absolutely free hand. They manned the municipal service up to the hilt with their own caste, and the result is a pitiably incompetent executive." Dr. Barry describes Poona as lying on a bed of sewage, with the wealthy quarters nearly as bad as the poor ones. In one especial quarter, where 30 deaths from plague occurred in a group of 54 houses, the inhabitants told him that they had represented their state to the municipality, but without avail. To the whole of Dr. Barry's long string of accusations there is practically no refutation offered, and only the feeblest excuses are offered for the horrible condition of things disclosed.

